# Influence of Fat Replacers on the Rheological, Tribological, and Aroma Release Properties of Reduced-Fat Emulsions

**DOI:** 10.3390/foods11060820

**Published:** 2022-03-12

**Authors:** Christopher N. Schädle, Solange Sanahuja, Stephanie Bader-Mittermaier

**Affiliations:** 1 Chair of Aroma and Smell Research, Department of Chemistry and Pharmacy, Friedrich-Alexander University Erlangen-Nürnberg, Henkestraße 9, 91054 Erlangen, Germany; 2 Department of Food Process Development, Fraunhofer Institute for Process Engineering and Packaging IVV, Giggenhauser Str. 35, 85354 Freising, Germany; stephanie.mittermaier@ivv.fraunhofer.de; 3 School of Agricultural, Forest and Food Sciences (HAFL), Bern University of Applied Sciences, Länggasse 85, 3052 Zollikofen, Switzerland; solange.sanahuja@bfh.ch

**Keywords:** lubricity, cheese aroma, dietary fiber, proton-transfer-reaction mass spectrometry (PTR-MS), corn dextrin, inulin, polydextrose, microparticulated whey protein, Nutriose, Simplesse

## Abstract

Reduced-fat food products can help manage diet-related health issues, but consumers often link them with poor sensory qualities. Thus, high-quality fat replacers are necessary to develop appealing reduced-fat products. A full-fat model emulsion was reduced in fat by replacing fat with either water, lactose, corn dextrin (CD), inulin, polydextrose, or microparticulated whey protein (MWP) as fat replacers. The effect of fat reduction and replacement, as well as the suitability of different types of fat replacers, were determined by analyzing fat droplet size distribution, composition, rheological and tribological properties, and the dynamic aroma release of six aroma compounds prevalent in cheese and other dairy products. None of the formulations revealed a considerable effect on droplet size distribution. MWP strongly increased the Kokini oral shear stress and viscosity, while CD exhibited similar values to the full-fat emulsion. All four fat replacers improved the lubricity of the reduced-fat samples. Butane-2,3-dione and 3-methylbutanoic acid were less affected by the changes in the formulation than butanoic acid, heptan-2-one, ethyl butanoate, and nonan-2-one. The aroma releases of the emulsions comprising MWP and CD were most similar to that of the full-fat emulsion. Therefore, CD was identified as a promising fat replacer for reduced-fat emulsions.

## 1. Introduction

Reduced-fat food products can contribute to tackling diet-related health issues, such as obesity, type 2 diabetes mellitus, cancer, and cardiovascular diseases [1]. The demand for such products is growing worldwide, especially in the fast-food and convenience sectors. Emulsions, mostly oil-in-water emulsions, are a common form of food with many applications in the fast-food industry, including sauces, mayonnaise, dressings, milk, and other dairy products. Dietary fat affects various properties in food emulsions, such as texture, creaminess, and flavor, and fat replacers must mimic the relevant properties in the reduced-fat products [2].

The use of high-quality fat replacers is one approach to developing appealing and tasty reduced-fat products. They can be carbohydrate-based, such as corn dextrin (CD), inulin (Inu), and polydextrose (Poly), or protein-based, such as microparticulated whey protein (MWP), typically exhibiting lower energy density (0–17.1 kJ/100 g, 0–4 kcal/100 g for the fat replacers) compared to fat (37 kJ/100 g, 9 kcal/100 g), as well as having a generally recognized as safe (GRAS) status, thus making them suitable for food applications [3,4]. Moreover, several studies have shown CD, Inu, Poly, and MWP to be promising fat replacers for emulsion-based product systems [3,5,6,7,8,9,10,11,12], although systematic studies are lacking, especially for CD, Inu, and Poly.

CD, Inu, and Poly are classified as water-soluble dietary fibers. CD is a partially hydrolyzed corn starch and thereby classified as resistant dextrin. Inu is a prebiotic fat replacer and belongs to the class of fructans. Poly is synthesized from glucose and sorbitol in the presence of citric acid and is used as a bulking agent to replace sugar or fat in food, as well as adding nutritional benefits by increasing satiety and exhibiting prebiotic effects [2,3,13]. Further, Poly can contribute to the mouthfeel and creaminess of reduced-fat products and act as a humectant [14]. By comparison, MWP is derived from whey protein via a heating and shearing process that forms spherical, uniform, and deformable protein particles in the size range of 0.1 to 3 µm, i.e., similar to fat droplets in milk and dairy emulsions [15,16]. The microparticulation process reduces the tendency of the MWP particles to aggregate and form gels upon heating.

The physicochemical and sensory properties of food emulsions depend to a large extent on the properties of the fat droplets, such as droplet size distribution, droplet charge, interfacial characteristics, physical state, and droplet concentration. While some properties are also influenced by other ingredients such as fat replacers, the droplet concentration depends solely on the fat concentration. In general, the viscosity of an oil-in-water emulsion decreases with decreasing fat droplet concentration. In order to compensate for the decreased viscosity of reduced-fat emulsions, thickeners such as starch or hydrocolloids in the aqueous phase can be used to increase the viscosity. Another approach is the use of non-fat particles such as MWP [17,18]. Ingredients used as fat replacers often have functional properties that mimic some of the attributes of fat such as mouthfeel, viscosity, and appearance [19]. Rheological methods can model parts of oral processing. However, the thin-film rheological behavior between the tongue and the palate can be described more adequately using tribological measurements. Tribology studies the friction of surfaces in relative motion [20]. While it has been increasingly performed in food science in recent years, there is still a significant research gap. A tribometer measures the coefficient of friction (COF), which is then displayed as a function of sliding speed in the so-called Stribeck curve. Several previous studies showed that the overall lubricity of the tribological system increases with increased fat content [21,22,23,24]. As a result, the friction decreases. Furthermore, it was also found that oral friction processes are important factors determining the sensory properties, thus tribological measurements have been related to in-mouth textural perception [18,25,26,27,28,29,30,31].

In addition to affecting textural and mouthfeel properties, fat reduction has a large influence on aroma release. The release rate of aroma compounds from the food matrix into the air depends on different factors governed by the properties of the food matrix and the aroma compounds. The thermodynamic and kinetic factors are determined by the resistance to mass transfer and the volatility of the aroma compounds, which are influenced by their hydrophobicity and enthalpy of vaporization. The mass transfer can be influenced by the viscosity of the food matrix and the interaction of aroma with non-volatile compounds in the food, such as lipids, protein, and carbohydrates [32].

Most aroma compounds are more soluble in lipids than in water due to their hydrophobic properties. Accordingly, the reduction and/or replacement of fat in reduced-fat products often causes changes in the aroma of the food [33]. In emulsions, the release of hydrophobic aroma compounds generally decreases with increased quantities of fat. A study by Frank et al. [34], for example, observed that hydrophobic compounds (heptan-2-one and ethyl butanoate) were released to a lesser degree when the fat content of the model emulsion decreased, whereas the release of hydrophilic aroma compounds (butane-2,3-dione or short-chain alcohols) remained largely unaffected [35]. Therefore, the use of fat replacers capable of binding aroma molecules is suggested to be desirable.

Interactions between carbohydrates—especially polysaccharides—and aroma compounds are often based on an increase in the viscosity [32] and a change in the diffusion coefficient of the aroma compounds in food. It is reported that carbohydrates in a solution induce a decrease in aroma release [33]. In contrast, smaller carbohydrates can structure water molecules in solutions and thus decrease the amount of free water, resulting in a salting-out of the aroma compounds and therefore increasing the aroma release. Polar compounds are more likely to be affected by the salting-out effect of low molecular weight carbohydrates than less polar compounds. On the other hand, polysaccharides may interact in various ways with aroma compounds at the molecular level, i.a., via hydrogen bonding, hydrophobic interactions, or molecular inclusion [36]. Some carbohydrates can form complexes with aroma compounds, as described for amylose [37]. Thereby, hydroxyl groups on the outside of the amylose helices can interact via hydrogen bonds with aroma compounds and aroma compounds can also be trapped inside the amylose helices through hydrophobic interactions, forming inclusion complexes [33].

Proteins are also known to bind and trap aroma compounds [38,39,40,41]. Proteins are often in a globular structure due to hydrophobic and electrostatic interactions, hydrogen bonding, van der Waals forces, and configurational entropy [42]. The interactions between proteins and aroma compounds are mostly reversible and include hydrophobic interactions as well as hydrogen bonding [43]. However, aroma compounds with carbonyl or thiol groups can bind irreversibly to proteins [44,45]. In emulsions, proteins can act as emulsifiers at the oil–water interface and additionally affect the release of hydrophobic aroma compounds in this way, whereas fat is more potent in retaining hydrophobic aroma compounds than proteins [32,46]. The hydrophobic interaction between the whey protein β-lactoglobulin and aroma compounds is one of the most-studied protein–aroma interactions [37]. Andriot et al. [47] described a higher retention of methyl ketones with increased β-lactoglobulin concentration, with a greater effect for nonan-2-one compared to heptan-2-one. The second-largest fraction of whey proteins, the α-lactalbumin, also binds aroma compounds such as ketones and aldehydes, but with a lower capacity than the other whey proteins [48]. In general, whey proteins have high aroma-binding capacities and their presence in dairy products is likely to lead to an unbalanced flavor, especially in reduced-fat or high-protein products [49]. The study of single whey protein components can elucidate individual interaction mechanisms. However, since whey protein usually consists of a mixture, it is more important for the industry to know the interaction of the mixture with the aroma compounds [50,51,52]. Moreover, while the interactions of whey protein and aroma compounds in aqueous solutions are well studied, much fewer studies have focused on the interaction of protein aggregates and microparticles such as MWP with aroma compounds in food [49].

A full-fat model emulsion was reduced in fat by replacing fat with either water, lactose, or a fat replacer (Inu, Poly, CD, or MWP). The full-fat model emulsion used in this study can be compared to cream, a dairy product containing around 30% fat. The effect of fat reduction and replacement was determined by analyzing fat droplet size distribution, composition, rheological and tribological properties, and the dynamic aroma release of six aroma compounds typical in various cheese and other dairy products [53,54,55,56,57]: butane-2,3-dione (diacetyl), butanoic acid (butyric acid), ethyl butanoate (ethyl butyrate), 3-methylbutanoic acid (isovaleric acid), heptan-2-one, and nonan-2-one. These differ in their chemical structure and properties, such as solubility and hydrophobicity (log *p*-value), and were determined by proton-transfer-reaction mass spectrometry (PTR-MS). PTR-MS analysis is widely used in food science to determine aroma release [58] and allows the real-time measurements of volatile aroma compounds with high sensitivity and without prior sample preparation.

The objective of the study was to gain new insights into the role of fat replacers in the development of appealing reduced-fat products, how fat replacers affect the emulsion properties, and how they interact with aroma compounds. While the effects of fat and fat reduction on the aroma release had been widely studied previously, only a few studies examined the effect of fat replacers and no studies are, to the best of our knowledge, available on the effect of the fat replacers Inu, Poly, CD, and MWP on the release of dairy and cheese aroma. Moreover, the reduced-fat samples were compared to the full-fat sample to determine the suitability of the used fat replacers in emulsion systems.

## 2. Materials and Methods

### 2.1. Materials

The model emulsions comprised deionized water (Wat), vegetable fat (Akoroma HM, refined, non-hydrogenated, 100% palm oil, solid fat content: 65% at 10 °C, 32% at 20 °C, 4% at 30 °C, and 1.5% at 35 °C, slip melting point: 30 °C from AAK AarhusKarlshamn AB, Malmö, Sweden), sodium edible casein (FN 5 S, minimum protein content of 88% and a maximum content of 6% moisture, 1.5% fat and 1% lactose from Rovita GmbH, Engelsberg, Germany), sunflower lecithin (Emulpur SF, de-oiled, powdered, moisture content of 1.1% from Cargill, Hamburg, Germany), and potassium sorbate (Sigma-Aldrich, Munich, Germany). Corn dextrin (CD) (Nutriose FM 06, partially acidic hydrolyzed corn starch having an average molecular weight of 5 kDa, 82 to 88% of dietary fibers, and a content of mono- and disaccharides of 0.3% and < 0.1% starch from Roquette Frères, Lestrem, France), inulin (Inu) (Orafti GR, average degree of polymerization ≤10, with 89% dietary fiber and maximum 8% sugar, from BENEO GmbH, Mannheim, Germany), microparticulated whey protein (MWP) (Simplesse 100, having a protein content of 54% and a particle size of 0.1 to 3 µm, from CP Kelco Germany GmbH, Großenbrode, Germany), and polydextrose (Poly) (Litesse Ultra, with a maximum content of reduced sugar of 0.25%, from DANISCO Deutschland GmbH, Niebüll, Germany) were used as fat replacers. Lactose (Lac) (pharmacopeia quality, DocMorris, Heerlen, Netherlands) was used as an inert filler [59] to validate the fat-mimicking properties of the fat replacers without changing the dry matter content of the emulsions.

The buffer solution was prepared from deionized water, disodium phosphate (anhydrous, 99.0%), and citric acid (anhydrous, 99.5%) (ChemSolute, Th. Geyer GmbH & Co. KG, Renningen, Germany).

The aroma compounds butane-2,3-dione (diacetyl), butanoic acid (butyric acid), and ethyl butanoate (ethyl butyrate) were obtained from Fluka (Steinheim, Germany), and 3-methylbutanoic acid (isovaleric acid), heptan-2-one, and nonan-2-one from Sigma-Aldrich (Taufkirchen, Germany). All aroma compounds had a purity of at least 98% or higher.

### 2.2. Preparation of the Model Emulsions

All emulsions were produced in the same manner but with their specific formulations (shown in Table 1). Potassium sorbate was mixed with the buffer solution, containing 50 mM citric acid solution added to a 68 mM disodium phosphate solution (to reach a pH of 5.7). For the reduced-fat emulsions, the reduced amount of fat was replaced in the formulation with Wat, Lac, or fat replacers (Inu, Poly, CD, or MWP, each individually), which were also added to the aqueous solution to ensure complete dissolution. The solution was subsequently hydrated for 30 min at 55 °C in a covered beaker, which simultaneously acted to completely melt the fat in another beaker. The emulsifiers, sodium casein and lecithin, were added to the liquid fat and mixed with an Ultra-Turrax (T25 digital, IKA-Werke GmbH & Co. KG, Staufen, Germany) for 1 min at 12,000 rpm. The aqueous solution was added to the fat with emulsifiers and homogenized for another 1 min using the Ultra-Turrax at 12,000 rpm. The aroma solutions were added to the coarse emulsion and homogenized for another 1 min at the same speed. To produce a fine and stable emulsion, the slurry was homogenized using a two-stage homogenizer (APV 2000, AxFlow, Düsseldorf, Germany) at 250 bar in the first stage and 50 bar in the second stage. The emulsions were stored at 1 °C until analysis. All emulsion samples were produced in batch sizes of 500 g and each formulation was produced at least three times. All measurements with exception of the compositional analysis were performed one day after preparation.

### 2.3. Preparation of the Aroma Solutions

Aroma mixtures were prepared as two separate solutions to account for the different solubilities of the selected aroma. First, a stock solution of each aroma compound was prepared in buffer solution (butane-2,3-dione, butanoic acid) or ethanol (3-methylbutanoic acid, ethyl butanoate, heptan-2-one, nonan-2-one) and subsequently, the stock solutions were diluted with buffer solution (aroma solution 1) or ethanol (aroma solution 2) to reach the desired concentrations (listed in Table 2). Next, 5 g of aroma solution 1 and 0.5 g of aroma solution 2 were added to the emulsion to reach the final concentrations in the 500 g sample. Critically, the concentration of ethanol in the emulsion did not exceed 1.6 µL∙g^−1^ and therefore did not present a limiting factor for the PTR-MS analyses (see later).

### 2.4. Compositional Analysis

The dry matter content of the emulsion samples was determined according to AOAC [60] with a thermo-gravimetrical system at 105 °C (TGA 701, Leco Instrumente GmbH, Mönchengladbach, Germany). The protein content was calculated based on the nitrogen content determined according to the Dumas combustion method, as described by AOAC [61] using a Nitrogen Analyzer TruMac N (Leco Instrumente GmbH, Mönchengladbach, Germany) and a conversion factor of N × 6.25. The fat content was determined based on the method of Caviezel, DGF C-III 19 (00) [62]. Additionally, the fats were derivatized with trimethylsulfonium hydroxide before the fatty acid contents were analyzed by gas chromatography. The pH was measured using a 206-pH2 digital pH meter (Testo SE and Co. KGaA, Lenzkirch, Germany).

### 2.5. Particle Size Distribution

The analysis of the droplet size of the fat droplets in the emulsions was carried out according to the method described by Senturk Parreidt et al. [63], using a long bench model MSS Mastersizer S, Software version 2.15, with an MS 1 small sample dispersion unit, and the 300 mm RF lens (Malvern Instruments Ltd., Malvern, UK). The droplet size was calculated using a polydispersity distribution analysis model and Mie theory with optical densities set at 1.33 for the wet phase and 1.53 for the disperse phase (Software Model: 3OHD). To arrange the sample concentration, the obscuration, which denotes the amount of laser light that has been lost by passing through the sample, was adjusted between 10% and 15%. The speed of the dispersion unit was set to 3000 min^−1^. The measurement was started after 2 min of dispersion and was repeated 1 min later to check for time-delayed effects, such as the agglomeration of the droplets. Example curves of the particle size distribution measurements can be found in the appendix (Figure S1). The mean values of the droplet sizes were calculated as the mean of at least nine measurements per sample. The particle size measurements are reported as d_v,0_._1_, d_v,0_._5_, d_v,0_._9_, d_3,2_, and d_4,3_. The diameters d_v,0_._1_, d_v,0_._5_, d_v,0_._9_ correspond to 10, 50, and 90 vol% on a relative cumulative droplet size curve, respectively. The Sauter mean diameter d_3,2_ is defined as the surface weighted mean diameter and is most sensitive to the presence of fine droplets in the size distribution. The De Brouckere mean diameter d_4,3_ is defined as the weighted mean volume diameter and is most sensitive to the presence of large particles in the size distribution [64]. An additional parameter to characterize the width of the size distribution is the span. The span of a volume-based size distribution is defined as Span = (d_v,0_._9_ − d_v,0_._1_)/d_v,0_._5_ and gives an indication of the distance between the 10% and 90% points, normalized to the midpoint.

### 2.6. Rheological Properties

The rheology of the emulsion samples was measured using a Physica MCR 301 controlled shear strain rotational rheometer (Anton Paar Germany GmbH, Ostfildern, Germany), with a concentric cylinder geometry (CC27, d = 27 mm). The system was held at 10 °C with a Peltier heating system (C-PTD200) after 13.5 mL of the emulsion was filled into the cup. A rest time of 3 min allowed additional loading stresses to dissipate. Flow curves were determined at 10 °C in three steps of 4 min duration by linearly increasing (0–200 s^−1^), holding (200 s^−1^), and linearly decreasing (200–0 s^−1^) the shear rate and measuring the shear stress. The rheological properties of the emulsion samples were characterized using the Herschel–Bulkley model (Equation (1)) for non-Newtonian fluids on step one, at increasing shear rate, because it is commonly used to describe the flow properties of emulsions [65]:

(1) $$\tau =\tau_{0}+K\cdot {\dot{\gamma }}^{n}$$

where τ is the shear stress (Pa), $\tau_{0}$ is the yield stress (Pa), $\dot{\gamma }$ is the shear rate (s^−1^), *K* is the consistency index (Pa∙s*^n^*), and *n* is the flow index (–). The apparent shear viscosity was determined at 10 s^−1^ and 100 s^−1^, in the range assumed to be representative of chewing and swallowing processes [66,67]. Moreover, a constant shear rate of 10 s^−1^ is considered to account for the perceived viscosity of semisolid products in the mouth [68]. Tárrega et al. [69] linked the viscosity at 10 s^−1^ and the mouthfeel for a vanilla desert, and Krzeminski et al. [70] used a shear rate of 100 s^−1^ to assess the oral perception of semisolid dairy products optimally [71]. The Kokini oral shear stress τ (Kokini OSS) was calculated from the Herschel–Bulkley parameters [72] (Equation (2)):

(2) $$\tau =\tau_{0}+Kv^{n}{\left(\frac{1}{h_{0}{}^{\frac{\left(n+1\right)}{n}}}+{\left(\frac{F}{R^{n+3}}\cdot \frac{n+3}{2\pi K}\right)}^{\frac{1}{n}}\cdot \frac{\left(n+1\right)t}{2n+1}\right)}^{\frac{n^{2}}{\left(n+1\right)}}$$

with the velocity of the tongue v = 2 cm∙s^−1^, normal force F = 1 N, radius of plug R = 2.5 cm, time t = 1 s, initial plug height $h_{0}$ = 0.2 cm, and the Herschel–Bulkley parameters yield stress $\tau_{0}$, consistency index K, and flow index n. The flow curve of step 1 obtained during increasing shear rate was pasted to the flow curve of step 3 obtained during decreasing shear rate to draw the hysteresis loop. The hysteresis area corresponds to $A=A_{up}-A_{down}$, where $A_{up}$ is the area under the flow curve of step 1 and $A_{down}$ of step 3 [69]. The area provides an indication of the time-dependent rheological effects of the emulsion samples. Example curves of the rheological measurements can be found in the appendix (Figure S2). Wall slippage or synaeresis were not observed during the three measurements of the individual sample batches.

### 2.7. Tribological Properties

The tribological properties of the emulsion samples were measured using the same rheometer described above, with a tribology measuring cell equipped with a Peltier temperature control (T-PTD200), Peltier hood (H-PTD200), measuring shaft (BC12.7), and sample holder (SH-BC6). A ball-on-three-pins set-up using a glass ball (soda-lime glass, 12.7 mm in diameter) and three polymeric pins (polydimethylsiloxane (PDMS), cylinder, 6 mm in height and diameter), the so-called tribo-pair, with a deflection angle of the pins of 45°, simulated the interaction of tongue and palate in the mouth [73]. An aliquot of 0.6 g emulsion was inserted in the cell at a constant temperature of 37 °C, which is the average temperature in the human mouth. The glass ball exerted a normal force of 7 N onto the pins. The force perpendicular to the surface of each pin was about 1.65 N, resulting from the normal force divided over three pins and with an angle of 45°. This value is between the in-mouth forces reported to be between 0.01 and 10 N [74]. Additional loading stresses were allowed to dissipate during a 5 min rest period. Then, the sliding speed was increased logarithmically from 6 × 10^−5^ mm∙s^−1^ to 1 × 10^3^ mm∙s^−1^ and the corresponding coefficient of friction (COF) was calculated from the friction force divided by the normal force. Three curves of COF versus sliding speed (referred to from hereon as curve 1, 2, and 3) were obtained without replacing the sample, and continuing with two sequences of 1 min resting followed by increasing the sliding speed, as mentioned above. The residues in the sample holder were removed carefully with water and a tissue between the measurements of the samples of the same sample batch. The sample holder and the pins were dried with compressed air, rinsed with ethanol, and again dried with compressed air before loading a new sample. Each sample batch was measured four times and with a new set of three PDMS pins for each batch.

### 2.8. Aroma Release Analysis by Proton-Transfer-Reaction Mass Spectrometry (PTR-MS)

The release dynamics of the six target aroma compounds from the emulsion samples were studied using a high-sensitivity proton-transfer-reaction mass spectrometer (hs-PTR-MS; IONICON Analytik GmbH, Innsbruck, Austria). Dynamic headspace analyses were carried out following the procedure reported by Siefarth et al. [75]. An aliquot of 50 g of the emulsion sample and a magnetic stirring bar were placed directly into a 180 mL perfluoroalkoxy (PFA) beaker (AHF Analysentechnik AG, Tübingen, Germany). The beaker was closed with an air-tight lid that contained two connection ports for headspace flushing and sampling. The beaker was placed on a magnetic stirrer (MIX 15 eco, 2 mag AG, Munich, Germany) in an incubator to stir the sample continuously during the measurement at 500 rpm. The temperature was held constantly at 37 °C to mimic body temperature. During the measurements, the headspace above the sample was flushed with humidified zero-air, i.e., air free of volatiles, which was supplied by a gas calibration unit (GCU-a, IONICON Analytik GmbH). The headspace was initially flushed at a flow rate of 1000 mL∙min^−1^ zero-air for 15 min, which was subsequently reduced to 150 mL∙min^−1^. The headspace gas from the beaker was sampled into the PTR-MS instrument at a flow rate of 45 mL∙min^−1^ throughout the measurements, with the excess gas being directed into a fume hood. The PTR-MS inlet and reaction chamber were held at 100 °C. A constant drift voltage of 600 V, as well as a reaction chamber pressure of 2.2 mbar, were set. The headspace gas was analyzed in multiple ion detection (MID) mode at the following mass-to-charge (*m*/*z*) ratios: *m*/*z* 87 (butane-2,3-dione), *m*/*z* 89 (butanoic acid), *m*/*z* 103 (3-methylbutanoic acid), *m*/*z* 115 (heptan-2-one), *m*/*z* 117 (ethyl butanoate), and *m*/*z* 143 (nonan-2-one), each measured at a dwell time of 10 s. These *m*/*z* ratios represent the predominant product ion *m*/*z* of the six aroma compounds, as determined in preliminary studies of each individual aroma compound. Additionally, primary ions at *m*/*z* 21 and 39 (protonated water isotope H_3_^18^O^+^ and its hydrate H_3_^18^O^+^ H_2_O) were each measured at a dwell time of 100 ms for the later normalization of the data, resulting in a duration of around 60 s for a complete measurement cycle. Data were analyzed for 5 min, starting 4 min after the adjustment of the flow rate to 150 mL∙min^−1^, thus for five complete cycles. The average headspace concentration was calculated over these five minutes for each aroma compound. A measurement of an empty beaker under the same conditions was used as a blank sample and subtracted from the average headspace concentration of the aroma compounds. The raw data were corrected for *m*/*z*-dependent detection efficiencies, according to instrument-specific transmission factors. Subsequently, these data were normalized by the means of the measured primary ions and drift pressure. The concentrations of the aroma compounds in the headspace were calculated with the sensitivity factor in Table 3. The sensitivity factors were determined for each aroma compound beforehand, using the curve gradient of the calibration curves determined by the use of a Liquid Calibration Unit (LCU, IONICON Analytik GmbH). Additionally, all aroma compounds were analyzed individually at the same settings in the scan mode (*m*/*z* 21 to 160) and the respective fragmentation spectrum was recorded. There was no significant overlap of the fragmentation spectra with the selected main *m*/*z* values (*m*/*z* 87, 89, 103, 115, 117, 143) for the evaluation of the six aroma compounds. Each formulation was also produced additionally without the aroma compounds and analyzed in PTR-MS for the presence of the six selected *m*/*z* ratios. No significant amounts of volatile components with the selected *m*/*z* ratios were found in any formulation. To estimate the specific heat capacity of the formulations, the temperature profile in the sample was recorded during the PTR-MS measurement in additional trials. No noteworthy differences occurred regarding the temperature course between the individual formulations. Therefore, it can be assumed that differences in the aroma release are not based on temperature differences between the samples. Each sample batch was measured three times.

### 2.9. Statistical Analysis

The Shapiro–Wilk test was used to confirm Gaussian distribution, and the Levene test was applied to test for the homogeneity of variance. The data were subsequently processed by a single-factor (univariate) analysis of variance (ANOVA) followed by Tukey’s honest significance test (α = 0.05). The data are expressed as the mean ± standard deviation. The Pearson correlation coefficient between the results was determined to evaluate possible correlations.

## 3. Results and Discussion

### 3.1. Composition

The full-fat sample and all samples with Lac or fat replacers (Inu, Poly, CD, MWP) exhibited a dry matter content between 32.4 and 33.6 g/100 g. The dry matter content of the reduced-fat emulsion with Wat was 18.3 ± 0.8 g/100 g. The absolute fat content was 30.0 ± 0.1 g/100 g in the full-fat emulsion and was approximately halved in the other samples. There was no significant difference in the absolute protein content (1.6–1.8 g/100 g) between all samples, with exception of the MWP sample. Solely, the fat replacer MWP contains protein and raised the absolute protein content to 9.8 ± 0.4 g/100 g.

The full-fat sample exhibited the highest energy density of around 1178 kJ/100 g (286 kcal/100 g). Depending on the fat replacer, the energy density of the reduced-fat samples was between 623 kJ/100 g (151 kcal/100 g) and 865 kJ/100 g (208 kcal/100 g), and thus was reduced by around 27% to 47% compared to the full-fat sample. All values for fat, protein, and dry matter content as well as the respective energy densities can be found in Table 4. The pH values of the samples were around pH 5.9 with no significant difference between them; only the sample with MWP had a significantly higher pH value with pH 6.4.

### 3.2. Particle Size Distribution

The metrics of the particle size distribution of all emulsion samples are shown in Table 5. All samples exhibited a unimodal distribution. The full-fat sample demonstrated a significantly larger fat droplet size for d_v,0_._1_. Nevertheless, the differences in d_v,0_._1_ droplet size were less than 0.09 µm, indicating a relatively similar fat droplet size in all samples for the small droplet portion of the particle size distribution. The median d_v,0_._5_ was lowest for the Lac sample with 0.76 ± 0.06 µm and around 0.80 µm for the Inu, Poly, and CD samples. The Wat sample showed a slightly larger droplet size of 0.90 ± 0.04 mm and the MWP sample showed an even larger size of 1.03 ± 0.09 µm. The significantly largest value was found for the full-fat sample. The d_v,0_._9_ value considers larger particles in the size distribution and was highest for the MWP sample (2.68 ± 0.22 µm), which was expected since the size range of MWP particles reaches up to 3 µm [15]. The De Brouckere mean diameter d_4,3_ is most sensitive to the presence of large particles and was also the largest for the MWP sample, indicating a slightly larger particle size for the protein particles of MWP compared to the fat droplets in the emulsion samples. However, based on the unimodal size distribution of the MWP samples, the MWP particles appear to be in the same size range as the fat droplets in the emulsion. According to Gu et al. [78], the Sauter mean diameter, d_32_, describes the mean diameter for most of the droplets in emulsions, and López-Castejón et al. [9] described a lower emulsifying capacity of emulsions with increasing d_32_. Our results showed significantly lower d_32_ values for all reduced-fat samples compared to the full-fat sample. However, due to very small differences in the d_3,2_ value, the emulsifying capacities of all samples were probably not affected by the fat droplet size.

### 3.3. Rheological Properties

Rheological measurements are important for the characterization of emulsions, especially to compare full-fat with reduced-fat food samples. The fat content is one of the main factors influencing the rheological properties of food emulsions [79]. The experimental data were fitted to the Herschel-Bulkley model (Equation (1)) as described previously, and yield stress, flow index, and consistency were calculated (Table 6).

**Figure 1:**
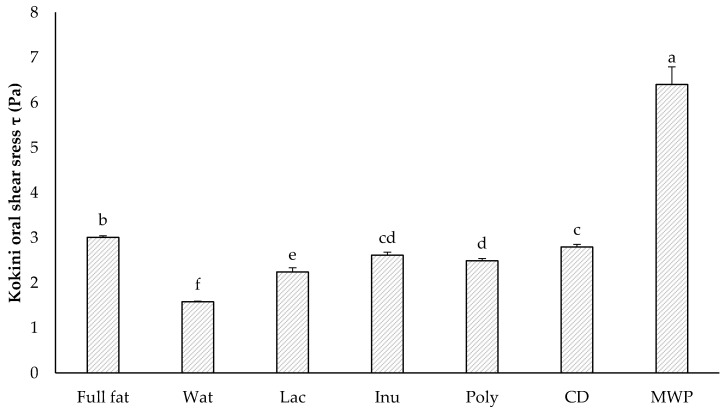
Kokini OSS of all emulsion samples. The data are expressed as the mean ± standard deviation (*n* = 9). Bars with different letters indicate significant differences between samples (*p* ≤ 0.05) following one-way ANOVA (Tukey).

All samples showed very low yield stress in the range from 8 to 20 mPa, and although significantly different, all samples could be considered as without yield stress due to the low values. The sample with MWP exhibited a significantly higher consistency compared to all other formulations, whereas no significant differences between all other formulations were found. For the emulsion samples Wat, Lac, Inu, Poly, and CD the flow index was around *n* = 1 and thus could be classified as Newtonian fluids. The full-fat sample with a flow index of 0.91 and the sample with MWP with a flow index of 0.75 showed shear-thinning properties. Additionally, solely the sample with MWP exhibited a hysteresis area, all other samples showed no time-dependent changes in the flow properties (data not shown). These properties of the MWP sample could be due to a breakdown of pre-existing MWP flocs in the emulsion due to a higher shear rate and longer shear time. Renard et al. [80] explained the shear-thinning and time-dependent (thixotropic) behavior of an MWP dispersion by means of this breakdown process. The process is also reversible due to rapidly reformed flocs after stopping the shear impact [80]. According to Liu et al. [81], the same flow behavior was found for microparticulated egg-white protein dispersions. The Wat sample exhibited a significantly lower viscosity compared to the full-fat sample, which was expected because of its significantly lower dry matter content. The use of Lac slightly increased the viscosity compared to the Wat sample, but only the use of the fat replacers Inu, Poly, or CD increased the viscosity at 10 s^−1^ to a degree that was not significantly different from the full-fat sample. Thus, the inclusion of fat replacers is mandatory for maintaining similar viscosities in reduced-fat emulsions. Several authors suggest that the viscosity at a shear rate of 10 s^−1^ and 100 s^−1^ is related to the perceived in-mouth thickness of semisolid products [66,67,68,69,70]. With a similar intention, Kokini et al. [82] proposed a physical index of in-mouth viscosity based on the change in shear stress during eating. Later, researchers such as Bayarri et al. [83] also found a high correlation between Kokini OSS and in-mouth thickness. Cook et al. [84] used it as a measure of in-mouth viscosity. The Kokini OSS was originally based on the Ostwald–de Waele model and was later extended by Stokes [72] to include yield stress, in order to fit the Kokini OSS (Equation (2)) to the Herschel-Bulkley model. The Kokini OSS was thus calculated from yield stress, consistency, and flow index, and is shown in Figure 1.

The MWP emulsion showed the highest Kokini OSS with more than twice the value of the full-fat sample, as well as the highest viscosity values, presumably due to the high water-holding capacity of MWP. The microparticulation process leaves few to no reactive sites (e.g., free thiol groups) to initiate cross-linking upon heating [12], which could have explained the high Kokini OSS values of the MWP emulsion. However, smaller MWP particles (<1 µm) are described to be able to interact with other proteins, such as caseins, resulting in enhanced gel strength and water-holding capacity [85]. This could be a possible explanation for the higher Kokini OSS value of the MWP emulsion. Due to a great span of 2.28 ± 0.03 µm for particles present in the MWP sample, it is clear that not only small particles are present in the emulsion. As the De Brouckere mean diameter, d_4,3_, is most sensitive to the presence of large droplets in the size distribution [64], large fat droplets in the full-fat and MWP samples were observed. Thus, the MWP and full-fat samples exhibited the highest Kokini OSS. The existence of larger droplets was associated with a higher viscosity of the continuous phase in emulsions and might be related to the lower emulsifying capacity of these systems [9]. Apart from that, no correlation was found between the particle size distribution and the Kokini OSS, per se (Figure S3). As expected, the Wat sample demonstrated the lowest Kokini OSS. All other samples also exhibited significantly lower Kokini OSS values compared to the full-fat sample. However, the difference between the full-fat and the CD sample was so small that it would probably not be detectable with human mouth perception, and therefore CD was deemed a suitable fat replacer to mimic fat in terms of the in-mouth viscosity of emulsions.

### 3.4. Tribological Properties

**Figure 2:**
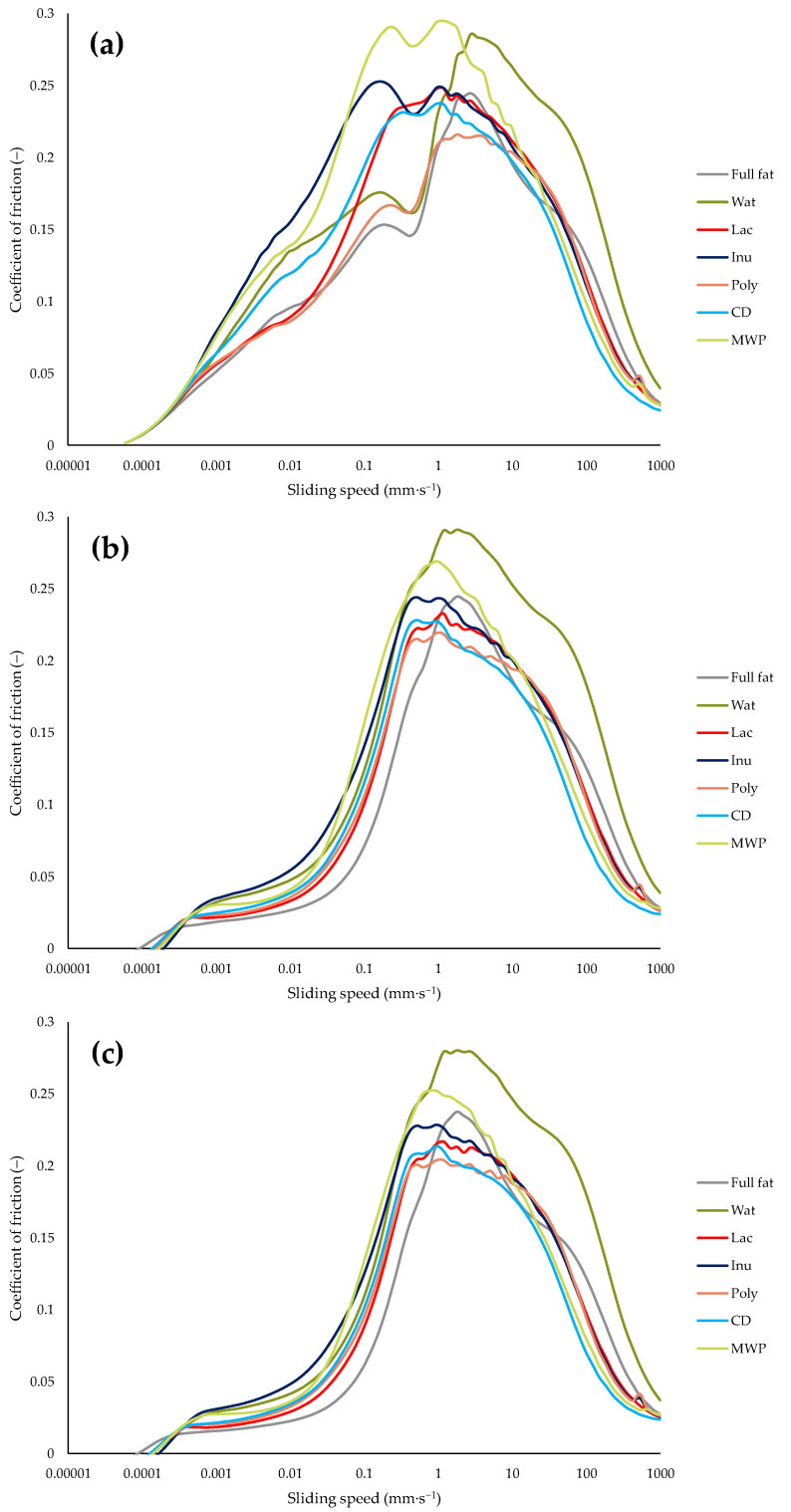
Stribeck curve 1 (**a**), curve 2 (**b**), and curve 3 (**c**): Coefficient of friction versus sliding speed of all emulsions. The curves are the mean values of all four measurements per each of the three batches (*n* = 12).

The tribological properties of the emulsion samples are described by the coefficient of friction (COF) versus the sliding speed, as shown in the Stribeck curves in Figure 2. The COF in food science indicates the lubrication properties of food products and is intended to describe the influence on the friction between tongue and palate in relative motion. The lower the COF, the better the lubrication properties of the food. All reduced-fat emulsions showed higher COF at lower sliding speeds (<1 mm∙s^−1^) compared to the full-fat sample. Interestingly, emulsions with MWP and Inu exhibited higher COF at lower sliding speeds, as shown in curve 1 (Figure 2a), compared to the other samples, but developed better lubrication properties at higher sliding speeds. In the static state and boundary friction regimes of the Stribeck curve, at low sliding speeds, the surfaces of the tribo-pair govern the friction, and the micro particles and possibly pre-existing flocs of MWP are assumed to act like a “spanner in the works” resulting in a higher COF. At increasing sliding speeds, in the so-called mixed friction regime, more food is carried into the gap, forcing the surfaces of the tribo-pair apart, and a lubricating film is formed. The flocs might have been disintegrated by the increasing rotary motion and the gap became wide enough for the MWP to act like a ball-bearing mechanism, reducing friction [21]. The theory on flocs destruction due to high-speed friction may also explain why the COF curves of MWP are more similar to the others in the second and third runs (curve 2 and 3; Figure 2b,c) after the emulsion had already been sheared in the first run (curve 1). Moreover, whereas fat reduces friction due to the formation of a fat film, the effect of the spherical MWP particles is proposed to reduce friction by reducing the contact area and changing the local relative motion from sliding to rolling [86,87]. As expected, the Wat sample with reduced fat and no added fat replacer showed the highest COF in the mixed friction regime. In general, a higher fat content was associated with better lubrication, hence a lower COF [88]. However, all samples with fat replacers exhibited lower COF in the mixed friction regime at increased sliding speeds compared to the full-fat sample, indicating better lubrication properties. Researchers suggested that a sliding speed range between 1 and 200 mm∙s^−1^ would describe mouth-like conditions during food consumption [89]. For example, at a sliding speed of 80 mm∙s^−1^, all reduced-fat samples with fat replacers showed significantly lower COF compared to the full-fat and Wat samples, whereas no significant differences were found between the samples with Inu, Poly, and Lac as well as between the full-fat and the sample with Lac. The best lubrication properties were determined for the CD sample, followed by the MWP sample.

In comparison with curve 2, the COF in curve 1 showed stronger variation between the formulations at sliding speeds in the boundary regime up to the peak COF. Moreover, the curves became smoother with fewer wavy lines. The emulsion may have been broken by mechanical forces during the first run and the fat could have plated-out and formed a fat film, which smoothed curves 2 and 3 of all formulations [21]. Almost all formulations showed a local minimum at a sliding speed of about 0.4 to 0.7 mm∙s^−1^ in curve 1. This could be an indication of the coalescence of the fat droplets at this time, forming a fat film in all samples [90]. Selway and Stokes [91] and Laguna et al. [92] studied semi-solid colloidal foods such as cream cheeses, yogurts, and custards as full- and low-fat samples. They also found a higher COF for the low-fat samples in the boundary regimes compared to the full-fat counterparts, despite identical viscosities. As in our study, the differences between the samples almost disappeared in the later hydrodynamic regimes, probably due to a coalesced film of fat [91,92,93]. Due to the fat film in our samples, the differences within one formulation across curves 1, 2, and 3 became smaller at sliding speeds above the local minimum. This fat film remains between the tribo-pair surfaces and, as previously mentioned, also smoothed curves 2 and 3 in the boundary regime. The MWP emulsion still showed a higher COF in the boundary regime of curves 2 and 3 due to the presence of particles, as explained above, but the difference is smaller. The order of the samples according to their COF in the mixed friction regime at higher sliding speeds is the same in all three curves, exhibiting a similar progression. In addition, curves 2 and 3 (Figure 2b,c) are quite similar over the entire sliding speed range and almost no differences occurred between these two measurement runs. Probably fewer than three tribological measurement runs are sufficient to evaluate the lubrication properties of these kinds of emulsion samples. Finally, no correlations between the COF at any sliding speed and Kokini OSS or viscosity were found in our study. In addition, no correlation was observed between the COF at any sliding speed and the metrics of the particle size distribution. For further investigation, we propose to stop the tribological measurement of the first run at different sliding speeds and analyze the treated sample with microscopic methods.

### 3.5. Aroma Release

The aroma release of the six aroma compounds was determined by dynamic headspace measurements in a specific time frame. The average headspace aroma concentration of each aroma compound in each formulation can be found in Table 7.

**Figure 3:**
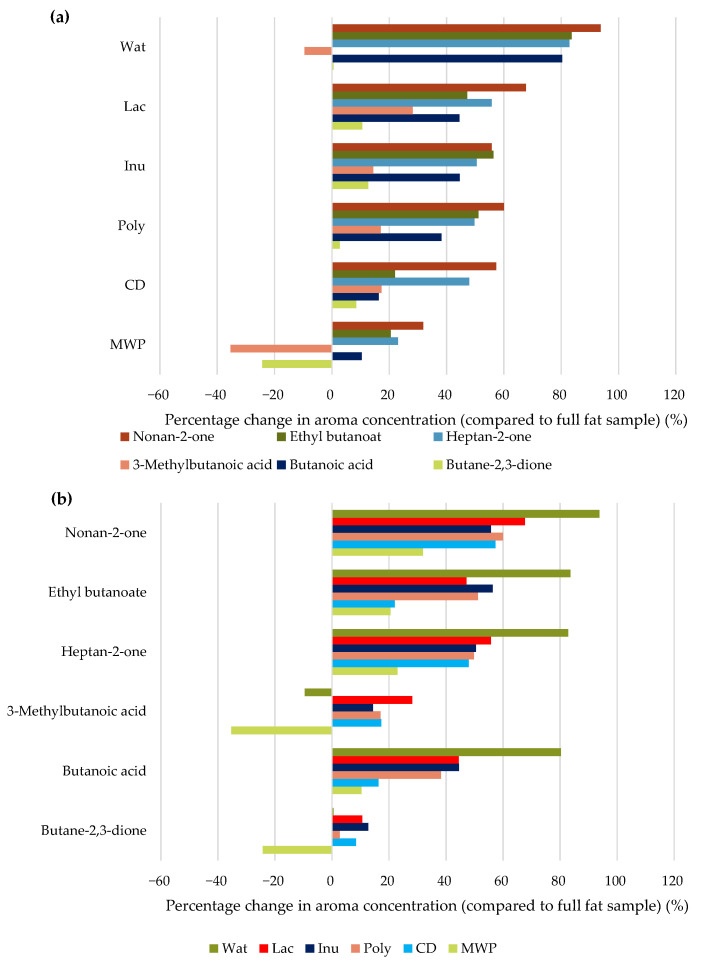
Percentage change in the headspace aroma concentration of all reduced-fat samples compared to the full-fat sample, sorted by emulsion formulation (**a**) and aroma compound (**b**).

To evaluate the influence of fat reduction and of the fat replacers on the aroma release, Figure 3 shows the percentage differences of each formulation compared to the full-fat sample. The data in Figure 3a is sorted by emulsion formulation and in Figure 3b by aroma compound. Positive values mean an enhanced aroma release and negative values a reduced aroma release compared to the full-fat sample, respectively. For a different view, the charts displaying the percentage changes relative to the Wat sample are also shown in the appendix (Figure S4).

**Figure 4:**
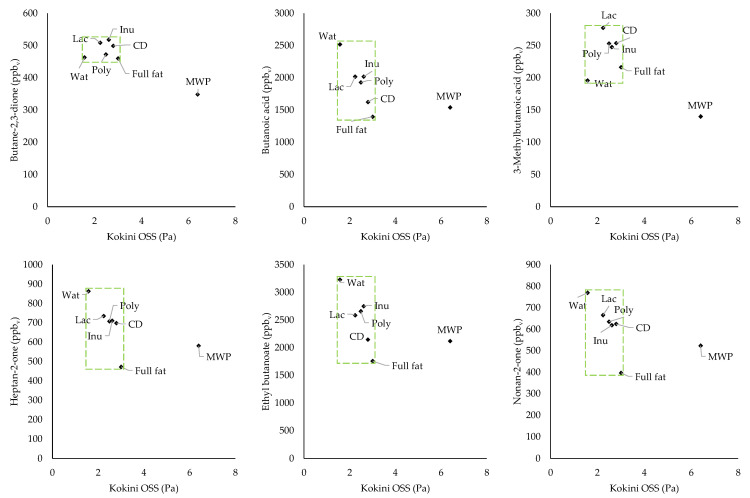
Headspace concentrations of the aroma compounds versus Kokini OSS.

Considering the aroma release of all aroma compounds for each formulation, the biggest changes in aroma release compared to the full-fat sample were found for the Wat sample. The smallest changes were found for the MWP and CD samples, indicating that the aroma profiles were most similar to the full-fat sample. In-between the Wat and the MWP/CD samples, the samples with Lac, Inu, and Poly showed a very similar aroma profile, indicating a similar influence on aroma release. Minor changes compared to the full-fat samples could mean no or little effect of the fat reduction and the use of fat replacers on the aroma release or similar aroma interaction capacities of the fat and the fat replacers. To get an indication of the effect on the aroma release, we can consider the differences between the full-fat and the Wat sample, in which we observed no significant changes in the aroma release of butane-2,3-dione and 3-methylbutanoic acid. Both aroma compounds are soluble in water and the reduction in fat had no effect on their release. However, the compounds butanoic acid, heptan-2-one, ethyl butanoate, and nonan-2-one exhibited a significantly higher aroma release from the Wat sample compared to the full-fat sample. The latter three aroma compounds are lipophilic and hardly soluble in water, which is linked to an increased aroma release with decreased fat content. Similar results were also described by Bayarri et al. [94], who found an increased aroma release for ethyl butanoate in model emulsions with different fat contents, and by Lauverjat et al. [95], who found an increased release of heptan-2-one from model cheeses. However, no effect or a reverse effect was observed in model cheeses for butane-2,3-dione, which is more soluble in water than in fat [32]. The higher aroma release of lipophilic aroma compounds with reduced fat content could be due to a higher concentration of these compounds in the fat phase, i.e., a higher concentration gradient. Interestingly, butanoic acid is miscible with water and has a lower log *p*-value than 3-methylbutanoic acid but also showed a significantly higher release from the Wat sample. This effect is likely due to the unbranched carbohydrate chain H_3_C-(CH_2_)*_n_*_>2_ in the molecules of the aroma compounds butanoic acid, heptan-2-one, ethyl butanoate, and nonan-2-one, which lead to hydrophobic interactions and a higher release from formulations with higher water contents. All aroma compounds exhibited a decreased aroma release from the MWP sample compared to the Wat sample, which could be linked to the significantly higher viscosity of the MWP sample or the binding of aroma compounds by proteins, both resulting in a slower diffusion of aroma compounds in the emulsion. Lesme et al. [96] also found a decreased aroma release for MWP compared to water, which, according to them, was due to the aroma-binding ability of proteins. Furthermore, they explained the differences in the release of various aroma compounds with the conformational state of proteins, since they used a native whey protein isolate and two types of MWP, one with a relatively small particle size (200 nm) and one with a polydisperse MWP (200 and 1000 nm). In addition, they used MWP in fat-free yogurts and found a decreased release of hydrophobic aroma compounds in particular, such as nonan-2-one, and attributed this to hydrophobic interactions of aroma compounds with proteins [96]. This suggests that in the present work, both high viscosity and interactions were responsible for the lower aroma release from the MWP sample. No correlation between the Kokini OSS, representing the in-mouth viscosity, and the release of the six aroma compounds was found (Figure 4), probably due to the small differences in viscosity between all samples with the exception of the MWP sample. Thus, only the clearly increased viscosity of the MWP sample had a significant influence on aroma release. Compared with the full-fat sample, the release of butane-2,3-dione and 3-methylbutanoic acid from the MWP sample was reduced. However, the release of the other four aroma compounds was slightly increased, although the viscosity of the MWP samples was also significantly higher than that of the full-fat sample. Thus, we conclude that the fat content appeared to have a bigger influence on the retention of lipophilic aroma compounds than the viscosity of the continuous aqueous phase in our emulsion samples.

Butane-2,3-dione is the only aroma compound in this study with a negative log *p*-value, indicating hydrophilic properties, and thus, it was least affected by the reduction of fat in the formulations. Moreover, the aroma release of butane-2,3-dione was least affected by changes in the formulation of the emulsions, followed by the aroma release of 3-methylbutanoic acid. Miettinen et al. [97] found a strong effect of the fat content on the release of the nonpolar compound linalool, but almost no effect on the more polar compound butane-2,3-dione. Butane-2,3-dione and 3-methylbutanoic acid reacted similarly to the changes in the formulation, as can be observed in the high correlation (*r* = 0.92) between the headspace concentrations of these two aroma compounds (Figure S5). The slightly higher release of these two aroma compounds from the reduced-fat samples with Lac, Inu, Poly, and CD compared to the full-fat sample could be explained by the ability of these ingredients to interact with water, resulting in a higher concentration of the aroma compounds in the remaining water due to a salting-out effect and thus a higher release due to a larger concentration gradient between the aqueous phase and the air. Delarue and Giampaoli [36] suggested that the salting-out effect enhances the volatility of polar compounds, whereas the headspace concentration of less-polar compounds tends to decrease. According to this, the salting-out effect probably only has an influence on butane-2,3-dione and 3-methylbutanoic acid, and the effect on the other aroma compounds is based on a different mechanism or interaction, respectively.

Excluding the MWP sample, the aroma release of butanoic acid, heptan-2-one, ethyl butanoate, and nonan-2-one differed for the other samples despite similar viscosities, indicating other effects of the ingredients than viscosity changes. These aroma compounds appeared to be affected very similarly by the changes in the formulations, with only slight deviations. The differences in the chemical structure between the four aroma compounds (butanoic acid, heptan-2-one, ethyl butanoate, and nonan-2-one) probably did not have a major impact on their release behavior. The Pearson correlation coefficients between the aroma release patterns of these four aroma compounds were between 0.90 and 1.00 (Figure S6), indicating a strong correlation supporting the hypothesis that they all behave in a very similar way. Despite their different chemical structure, they all exhibited lipophilic properties and different capacities of H-bridge formations and hydrophobic interactions between these four aroma compounds, and the fat replacers could be the reason for the different releases between the formulations. In general, the volatility of aroma compounds decreases with an increase in fat droplet size in emulsions [35,97,98]. However, in our study, no correlations were found, which can likely be attributed to the very similar fat droplet size distribution in all emulsion samples.

It should be considered that dynamic headspace measurements are suitable to analyze aroma–matrix interactions and release behaviors, but for the prediction of the perceived aroma in the mouth, other aspects should be taken into account. Roberts et al. [99] compared the headspace, nosespace, and sensory intensity ratings of aroma compounds. Overall, all methods determined a lower release of lipophilic aroma compounds as the fat content increased. However, they described an overestimation using headspace instead of nosespace methods to determine the aroma absorption by fat [99]. The in-mouth aroma release could happen from thin liquid layers, and the reservoir of the fat phase is not as important as in the bulk phase, unlike in headspace measurements [100], resulting in a high aroma concentration gradient between the thin liquid film and the air-flow of the breath [83]. This concentration gradient leads to an increased aroma release. Moreover, the researchers Bayarri et al. [94] and Bylaite et al. [101] did not find any differences in the aroma release from samples with different viscosities, but nevertheless observed differences in the aroma perception in sensory tests, suggesting that viscosity may influence in-mouth perception [94]. However, they all used static headspace measurements and compared them to dynamic nosespace measurements or sensory testing, which could explain the differences. Furthermore, oral physiology parameters and the presence of saliva had an influence on the in-mouth aroma release [46] and should be considered in further studies.

## 4. Conclusions

Full-fat and reduced-fat model emulsions with Wat, Lac, or different fat replacers (Inu, Poly, CD, MWP) were investigated, and characteristics including the fat droplet distribution, rheological and tribological properties, and aroma release of six different aroma compounds were analyzed. Fat reduction and the fat replacers only had a minor impact on the fat droplet size and the impact of the fat droplet size on the emulsion properties was negligible. The fat reduction had a significant effect on the viscosity and Kokini OSS of the emulsions. The fat replacers Inu, Poly, and CD neutralized this effect. They apparently are suitable mimics of the rheological properties of fat in emulsions. The fat replacer MWP increased viscosity and Kokini OSS to an order of magnitude higher value than the full-fat sample. The CD sample demonstrated the best friction-lowering properties at typical sliding speeds during oral processing, followed by the MWP sample. Both proved suitable to mimic the general lubrication properties of fat in emulsion systems. The aroma release was influenced by fat reduction rather than viscosity. The samples with Lac, Inu, and Poly behaved very similarly in their influence on aroma release. The aroma release from the MWP and the CD sample came closest to the full-fat sample among all reduced-fat samples. The aroma release of butane-2,3-dione and 3-methylbutanoic acid was least influenced by changes in the formulations, whereas the release of the lipophilic aroma compounds butanoic acid, heptan-2-one, ethyl butanoate, and nonan-2-one was significantly increased by fat reduction. In conclusion, it is not sufficient to adapt the dry matter content using, for example, lactose to obtain satisfactory products with properties comparable to the full-fat product. MWP did provide good results in aroma release and tribological measurements but had a strong impact on the rheological properties, resulting in emulsions with significantly increased viscosity. The use of CD provided emulsions with a similar in-mouth viscosity and aroma release, as well as improved lubrication properties, making it a suitable and promising fat replacer in emulsion systems. Further rheological, tribological, and aroma release studies should consider additional oral conditions, such as the presence of saliva and physiology parameters, and include human sensory studies.

## Figures and Tables

**Table 1:** Formulations of the emulsions (Wat = water, Lac = lactose, Inu = inulin, Poly = polydextrose, CD = corn dextrin, MWP = microparticulated whey protein).

	Full Fat	Wat	Lac	Inu	Poly	CD	MWP
	(g/100 g)	(g/100 g)	(g/100 g)	(g/100 g)	(g/100 g)	(g/100 g)	(g/100 g)
Buffer solution	65.7	80.7	65.7	65.7	65.7	65.7	65.7
Fat	30	15	15	15	15	15	15
Lac/Inu/Poly/CD/MWP	0	0	15	15	15	15	15
Sodium casein	2	2	2	2	2	2	2
Sunflower lecithin	1	1	1	1	1	1	1
Aroma solution 1	1	1	1	1	1	1	1
Aroma solution 2	0.1	0.1	0.1	0.1	0.1	0.1	0.1
Potassium sorbate	0.2	0.2	0.2	0.2	0.2	0.2	0.2

**Table 2:** Concentrations of aroma compounds in aroma solutions 1 and 2 and the resulting final concentrations in the emulsion.

Aroma Solution	Aroma Compound	Concentration in Aroma Solution (µg∙g^−1^)	Concentration in Emulsion (µg∙g^−1^)
1	Butane-2,3-dione	243	2.4
	Butanoic acid	8484	84.8
	3-Methylbutanoic acid	24,097	241.0
	Ethyl butanoate	1514	15.1
2	Heptan-2-one	5237	5.2
	Nonan-2-one	56,923	56.9

**Table 3:** Aroma compounds and their physicochemical properties.

Aroma Compound	Molecular Weight	Major Product Ion	Sensitivity Factor	Log *p*-Value [76]	Odor Quality [77]	CAS
	(g∙mol^−1^)	(*m*/*z*)	(ncps∙ppb_v_^−1^)			
Butane-2,3-dione	86	87	3.98	−1.34	Buttery	431-03-8
Butanoic acid	88	89	1.64	0.79	Rancid, cheesy, sharp	107-92-6
3-Methylbutanoic acid	102	103	1.81	1.16	Sharp, sweaty, sweet, fruity	503-74-2
Ethyl butanoate	116	117	0.67	1.71	Pineapple, banana, fruity	105-54-4
Heptan-2-one	114	115	2.86	1.98	Blue cheese, fruity, sweet	110-43-0
Nonan-2-one	142	143	3.91	3.16	Fruity, musty, rose, tea-like	821-55-6

**Table 4:** Composition of the samples based on 100 g emulsion (abs = absolute, DM = dry matter, Lac = lactose, Inu = inulin, Poly = polydextrose, CD = corn dextrin, MWP = microparticulated whey protein).

Formulation	DM	Fat (abs)	Fat (in DM)	Protein (abs)	Protein (in DM)	Energy Density ^1^	Energy Density ^1^
per 100 g	(g)	(g)	(g)	(g)	(g)	(kJ)	(kcal)
Full fat	33.6 ± 0.3 ^a^	30.0 ± 0.1 ^a^	89.3 ± 0.9 ^a^	1.6 ± 0.1 ^b^	4.8 ± 0.4 ^c^	1178	286
Wat	18.3 ± 0.8 ^b^	15.8 ± 0.5 ^c^	86.5 ± 6.0 ^a^	1.7 ± 0.1 ^b^	9.2 ± 0.9 ^b^	623	151
Lac	32.4 ± 0.3 ^a^	17.8 ± 0.4 ^bc^	54.9 ± 0.7 ^bc^	1.8 ± 0.0 ^b^	5.6 ± 0.1 ^c^	865	208
Inu	33.1 ± 0.7 ^a^	15.7 ± 0.4 ^c^	47.6 ± 1.1 ^c^	1.7 ± 0.2 ^b^	5.2 ± 0.6 ^c^	698	169
Poly	33.1 ± 0.1 ^a^	17.0 ± 0.6 ^bc^	51.4 ± 1.6 ^bc^	1.8 ± 0.0 ^b^	5.3 ± 0.1 ^c^	623	151
CD	32.5 ± 0.2 ^a^	18.3 ± 2.2 ^bc^	56.2 ± 7.0 ^bc^	1.8 ± 0.0 ^b^	5.5 ± 0.0 ^c^	748	181
MWP	32.4 ± 0.1 ^a^	19.1 ± 0.5 ^b^	58.8 ± 1.6 ^b^	9.8 ± 0.4 ^a^	30.2 ± 1.2 ^a^	863	208

^1^ Energy density was calculated using the manufacturer specifications of the ingredients and the respective formulations of the samples. The data are expressed as the mean ± standard deviation (*n* = 9). Values followed by different letters in a column indicate significant differences between samples (*p* ≤ 0.05) following one-way ANOVA (Tukey).

**Table 5:** Metrics of the particle size distribution for all emulsion samples.

Formulation	d_v,0_._1_	d_v,0_._5_	d_v,0_._9_	d_4,3_	d_3,2_	Span
	(µm)	(µm)	(µm)	(µm)	(µm)	(µm)
Full fat	0.38 ± 0.01 ^a^	1.09 ± 0.06 ^a^	2.46 ± 0.21 ^b^	1.28 ± 0.09 ^a^	0.78 ± 0.03 ^a^	1.90 ± 0.09 ^e^
Wat	0.33 ± 0.02 ^b^	0.90 ± 0.04 ^c^	2.15 ± 0.11 ^cd^	1.10 ± 0.05 ^b^	0.67 ± 0.04 ^c^	2.02 ± 0.06 ^d^
Lac	0.32 ± 0.01 ^c^	0.76 ± 0.06 ^e^	1.79 ± 0.18 ^e^	0.93 ± 0.09 ^d^	0.61 ± 0.03 ^d^	1.93 ± 0.10 ^e^
Inu	0.30 ± 0.02 ^d^	0.80 ± 0.05 ^d^	2.23 ± 0.25 ^c^	1.08 ± 0.08 ^b^	0.61 ± 0.04 ^d^	2.41 ± 0.19 ^a^
Poly	0.30 ± 0.01 ^d^	0.81 ± 0.05 ^d^	2.05 ± 0.15 ^d^	1.03 ± 0.07 ^c^	0.61 ± 0.03 ^d^	2.14 ± 0.07 ^c^
CD	0.31 ± 0.01 ^cd^	0.78 ± 0.03 ^de^	1.88 ± 0.09 ^e^	0.96 ± 0.04 ^d^	0.61 ± 0.02 ^d^	2.03 ± 0.07 ^d^
MWP	0.34 ± 0.02 ^b^	1.03 ± 0.09 ^b^	2.68 ± 0.22 ^a^	1.31 ± 0.11 ^a^	0.72 ± 0.05 ^b^	2.28 ± 0.03 ^b^

The data are expressed as the mean ± standard deviation (*n* ≥ 27). Values followed by different letters in a column indicate significant differences between samples (*p* ≤ 0.05) following one-way ANOVA (Tukey).

**Table 6:** Viscosity at a shear rate of 10 s^−1^ and 100 s^−1^ as well as yield stress, consistency, and flow index (Herschel-Bulkley model) of the rheological characterization of the emulsion samples.

Formulation	Viscosity η at 10 s^−1^ (mPa∙s)	Viscosity η at 100 s^−1^ (mPa∙s)	Yield Stress τ_0_ (mPa)	Consistency K (mPa∙s*^n^*)	Flow Index *n* (–)
Full fat	25.7 ± 0.9 ^b^	20.5 ± 0.1 ^b^	20.0 ± 3.0 ^a^	30.5 ± 1.7 ^b^	0.91 ± 0.01 ^d^
Wat	5.7 ± 0.1 ^d^	5.6 ± 0.1 ^e^	15.0 ± 0.6 ^bc^	4.3 ± 0.1 ^b^	1.05 ± 0.00 ^a^
Lac	12.2 ± 1.3 ^cd^	11.6 ± 1.0 ^d^	16.0 ± 5.6 ^ab^	10.5 ± 1.1 ^b^	1.02 ± 0.01 ^b^
Inu	16.6 ± 0.9 ^bc^	15.6 ± 0.8 ^cd^	11.4 ± 2.6 ^cd^	16.2 ± 1.3 ^b^	0.99± 0.01 ^c^
Poly	15.0 ± 0.6 ^bcd^	14.2 ± 0.5 ^cd^	13.3 ± 2.1 ^bc^	14.1 ± 0.8 ^b^	1.00 ± 0.00 ^c^
CD	18.9 ± 0.9 ^bc^	18.0 ± 0.7 ^bc^	8.1 ± 2.6 ^d^	18.3 ± 1.4 ^b^	0.99 ± 0.01 ^c^
MWP	127.6 ± 21.1 ^a^	79.2 ± 8.82 ^a^	not detected	260.2 ± 66.0 ^a^	0.75 ± 0.02 ^e^

The data are expressed as the mean ± standard deviation (*n* = 9). Values followed by different letters in a column indicate significant differences between samples (*p* ≤ 0.05) following one-way ANOVA (Tukey).

**Table 7:** Headspace concentration of aroma compounds in different emulsion formulations.

Formulation	Butane-2,3-dione (ppb_v_)	Butanoic Acid (ppb_v_)	3-Methylbutanoic Acid (ppb_v_)	Heptan-2-one (ppb_v_)	Ethyl Butanoate (ppb_v_)	Nonan-2-one (ppb_v_)
Full fat	459.6 ± 21.1 ^c^	1384.4 ± 114.7 ^d^	216.1 ± 10.1 ^c^	472.2 ± 22.8 ^d^	1758.0 ± 141.1 ^d^	397.0 ± 19.0 ^d^
Wat	462.4 ± 26.7 ^c^	2514.9 ± 392.1 ^a^	195.6 ± 9.6 ^c^	863.5 ± 72.7 ^a^	3228.9 ± 550.0 ^a^	769.6 ± 73.0 ^a^
Lac	508.4 ± 12.6 ^a^	2014.8 ± 297.1 ^b^	277.1 ± 35.3 ^a^	735.2 ± 47.9 ^b^	2587.3 ± 402.3 ^bc^	665.8 ± 81.3 ^b^
Inu	517.9 ± 25.3 ^a^	2016.5 ± 105.7 ^b^	247.3 ± 10.3 ^b^	710.7 ± 32.7 ^b^	2749.9 ± 220.8 ^b^	618.6 ± 27.9 ^b^
Poly	472.2 ± 18.4 ^bc^	1927.4 ± 118.5 ^bc^	253.0 ± 12.5 ^b^	707.4 ± 57.4 ^b^	2658.1 ± 212.1 ^b^	635.2 ± 68.6 ^b^
CD	498.6 ± 21.7 ^ab^	1621.9 ± 245.0 ^cd^	253.5 ± 7.6 ^ab^	698.4 ± 30.7 ^b^	2145.0 ± 235.4 ^cd^	624.5 ± 22.2 ^b^
MWP	347.9 ± 31.4 ^d^	1539.0 ± 195.6 ^d^	139.8 ± 12.0 ^d^	580.9 ± 88.7 ^c^	2118.7 ± 295.3 ^d^	523.6 ± 104.1 ^c^

The data are expressed as the mean ± standard deviation (*n* = 9). Values followed by different letters in a column indicate significant differences between samples (*p* ≤ 0.05) following one-way ANOVA (Tukey).

## Data Availability

The data presented in this study is available on request from the corresponding author.

## Supplementary Materials

The following supporting information can be downloaded at: https://www.mdpi.com/article/10.3390/foods11060820/s1, Figure S1: Particle size distribution of four single measurements as example curves for the particle size determination., Figure S1: Flow curve diagram for single measurements of all emulsion formulations as example curves for rheological analysis (arrows indicate curve progression for MWP sample), Figure S3: Characteristic numbers of the particle size distribution versus Kokini OSS of all emulsions, Figure S4: Percentage change in the headspace aroma concentration of all emulsion samples compared to the Wat sample, sorted by emulsion formulation (a) and by aroma compound (b), Figure S5: Correlation between the headspace concentrations of the aroma compounds butane-2,3-dione and 3-methylbutanoic acid in the different emulsions (r = correlation coefficient), Figure S6: Correlations between the headspace concentration of the aroma compounds butanoic acid, ethyl butanoate, heptan-2-one, and nonan-2-one in the different emulsions (r = correlation coefficient).
