# Supplementary material for: Influence of Fat Replacers on the Rheological, Tribological, and Aroma Release Properties of Reduced-Fat Emulsions
Source: Foods. 2022 Mar 12;11(6):820. doi: 10.3390/foods11060820 (PMC8947701; doi:10.3390/foods11060820)
Supplement: Supplementary file 1 [file foods-11-00820-s001.zip › foods-1622865-Supplementary.pdf]

Supplementary Materials

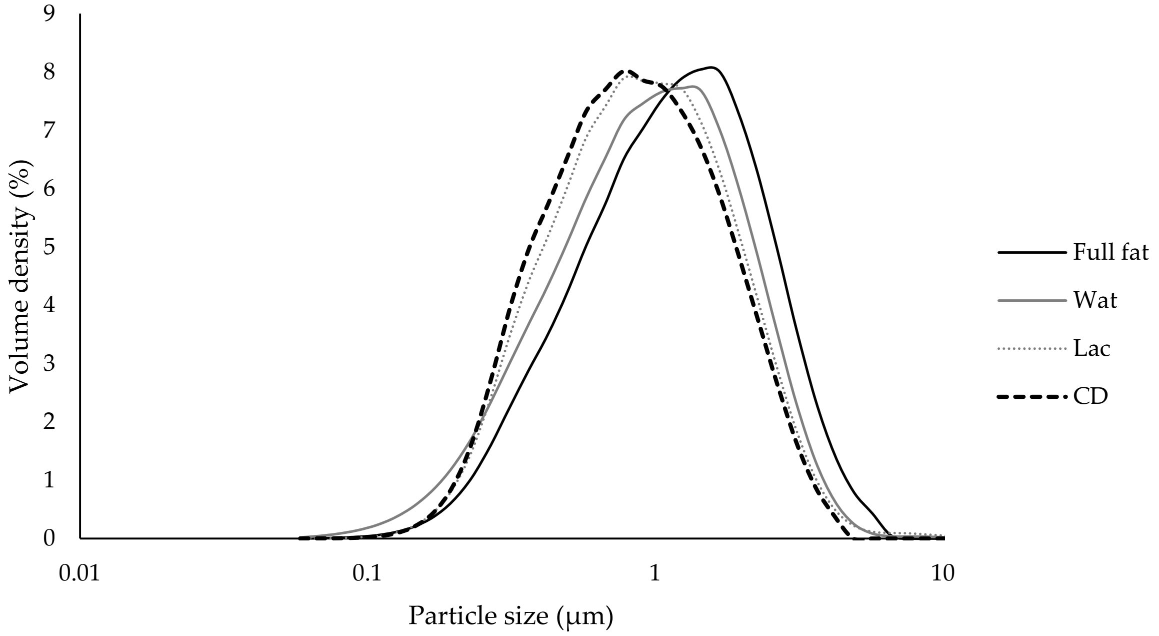

Figure S1. Particle size distribution of four single measurements as example curves for the particle size determination.

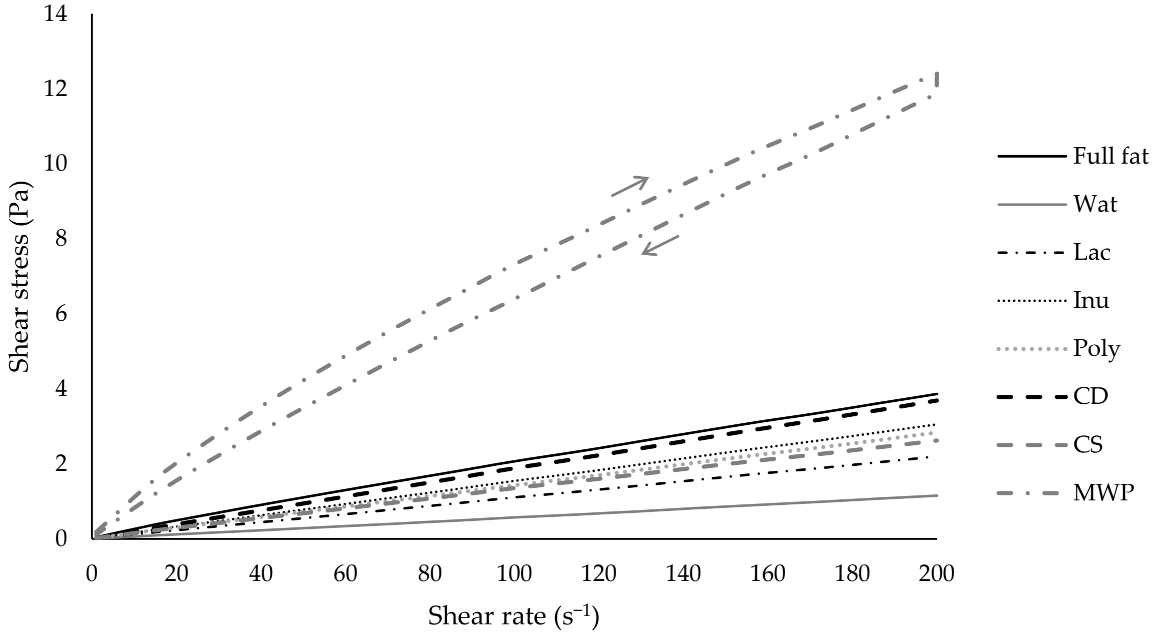

Figure S2. Flow curve diagram for single measurements of all emulsion formulations as example curves for rheological analysis (arrows indicate curve progression for MWP sample).

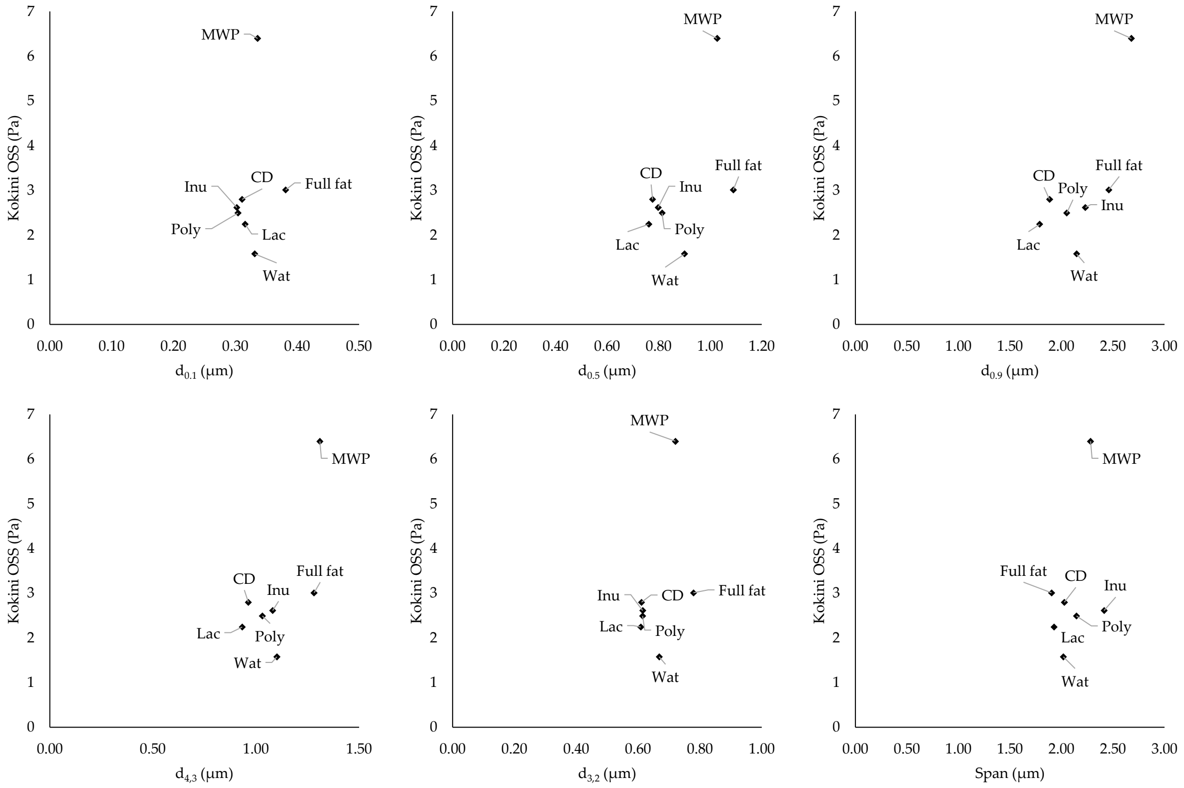

Figure S3. Characteristic numbers of the particle size distribution versus Kokini OSS of all emulsions.

(a)

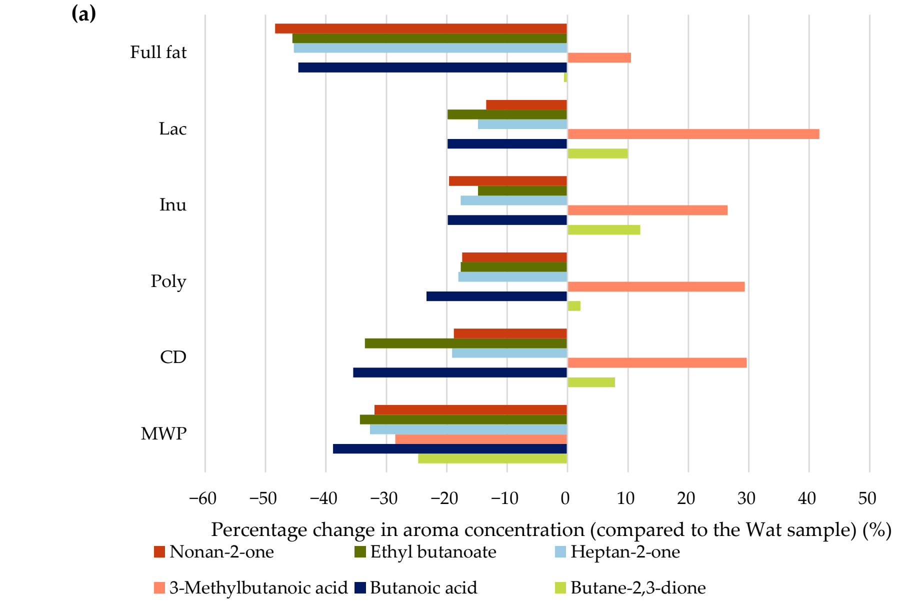

(b)

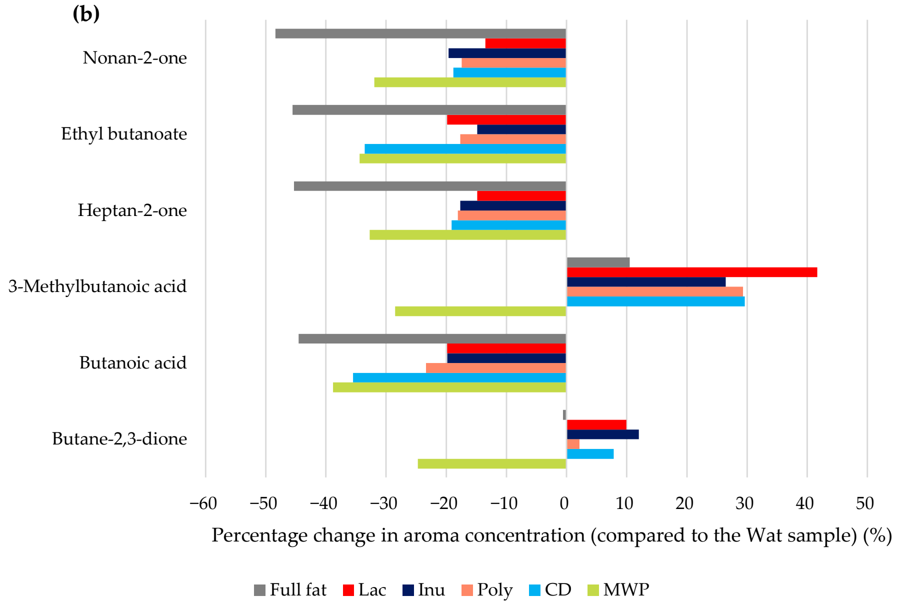

Figure S4. Percentage change in the headspace aroma concentration of all emulsion samples compared to the Wat sample, sorted by emulsion formulation (**a**) and by aroma compound (**b**).

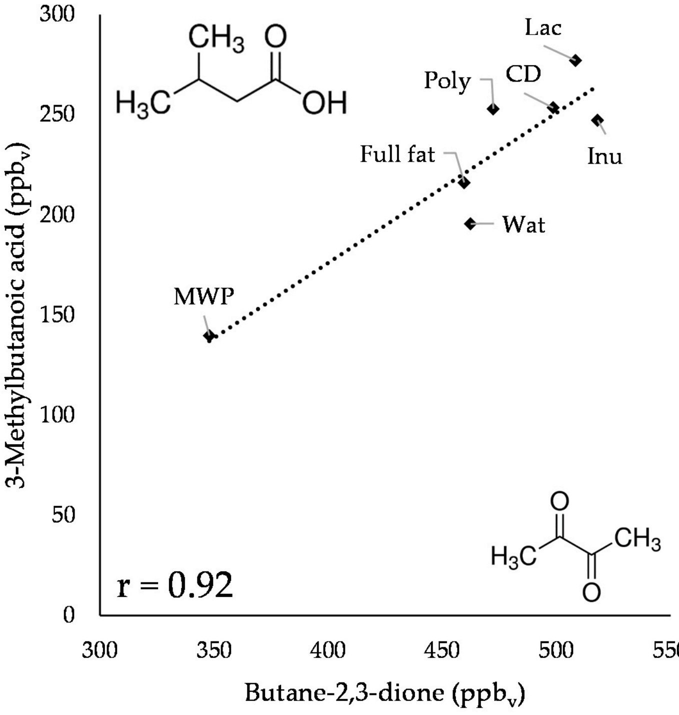

Figure S5. Correlation between the headspace concentrations of the aroma compounds butane-2,3-dione and 3-methylbutanoic acid in the different emulsions (*r* = correlation coefficient).

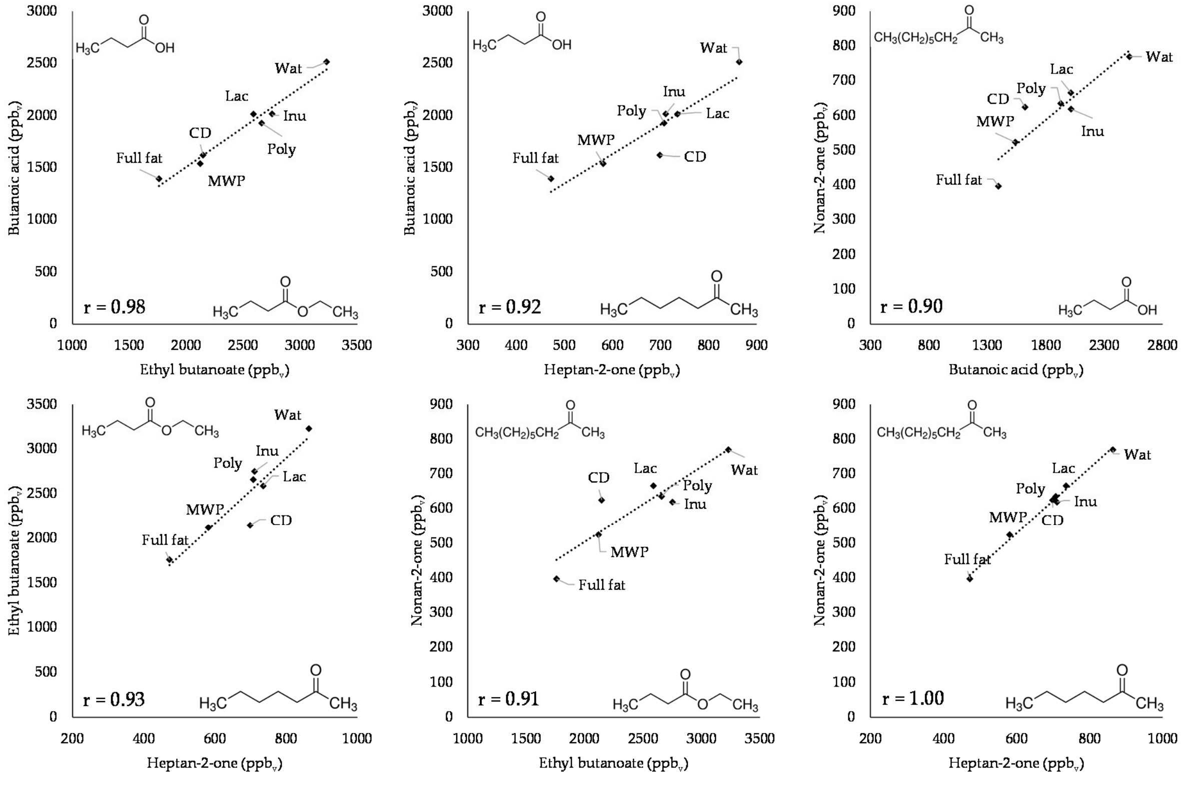

Figure S6. Correlations between the headspace concentration of the aroma compounds butanoic acid, ethyl butanoate, heptan-2-one, and nonan-2-one in the different emulsions (*r* = correlation coefficient).
